# SARS-CoV-2, influenza A/B and respiratory syncytial virus positivity and association with influenza-like illness and self-reported symptoms, over the 2022/23 winter season in the UK: a longitudinal surveillance cohort

**DOI:** 10.1186/s12916-024-03351-w

**Published:** 2024-03-26

**Authors:** Elisabeth Dietz, Emma Pritchard, Koen Pouwels, Muhammad Ehsaan, Joshua Blake, Charlotte Gaughan, Eric Haduli, Hugh Boothe, Karina-Doris Vihta, Tim Peto, Nicole Stoesser, Philippa Matthews, Nick Taylor, Ian Diamond, Ruth Studley, Emma Rourke, Paul Birrell, Daniela De Angelis, Tom Fowler, Conall Watson, David Eyre, Thomas House, Ann Sarah Walker

**Affiliations:** 1 Nuffield Department of Medicine, University of Oxford, Oxford, UK; 2 The National Institute for Health Research Health Protection Research Unit in Healthcare Associated Infections and Antimicrobial Resistance at the University of Oxford, Oxford, UK; 3 Health Economics Research Centre, Nuffield Department of Population Health, University of Oxford, Oxford, UK; 4 Berkshire and Surrey Pathology Services, Camberley, UK; 5 MRC Biostatistics Unit, University of Cambridge, Cambridge, UK; 6 Office for National Statistics, Newport, UK; 7 Department of Engineering, University of Oxford, Oxford, UK; 8 Department of Infectious Diseases and Microbiology, Oxford University Hospitals NHS Foundation Trust, John Radcliffe Hospital, Oxford, UK; 9 The Francis Crick Institute, 1 Midland Road, London, UK; 10 Division of Infection and Immunity, University College London, London, UK; 11 UK Health Security Agency, London, UK; 12 William Harvey Research Institute, Queen Mary University of London, London, UK; 13 Big Data Institute, Nuffield Department of Population Health, University of Oxford, Oxford, UK; 14 University of Manchester, Manchester, UK; 15 The National Institute for Health Research Oxford Biomedical Research Centre, University of Oxford, Oxford, UK

**Keywords:** SARS-CoV-2, Respiratory syncytial virus, Influenza a/b, Influenza-like illness, Surveillance, Symptoms, Vaccination

## Abstract

**Background:**

Syndromic surveillance often relies on patients presenting to healthcare. Community cohorts, although more challenging to recruit, could provide additional population-wide insights, particularly with SARS-CoV-2 co-circulating with other respiratory viruses.

**Methods:**

We estimated the positivity and incidence of SARS-CoV-2, influenza A/B, and RSV, and trends in self-reported symptoms including influenza-like illness (ILI), over the 2022/23 winter season in a broadly representative UK community cohort (COVID-19 Infection Survey), using negative-binomial generalised additive models. We estimated associations between test positivity and each of the symptoms and influenza vaccination, using adjusted logistic and multinomial models.

**Results:**

Swabs taken at 32,937/1,352,979 (2.4%) assessments tested positive for SARS-CoV-2, 181/14,939 (1.2%) for RSV and 130/14,939 (0.9%) for influenza A/B, varying by age over time. Positivity and incidence peaks were earliest for RSV, then influenza A/B, then SARS-CoV-2, and were highest for RSV in the youngest and for SARS-CoV-2 in the oldest age groups. Many test positives did not report key symptoms: middle-aged participants were generally more symptomatic than older or younger participants, but still, only ~ 25% reported ILI-WHO and ~ 60% ILI-ECDC. Most symptomatic participants did not test positive for any of the three viruses. Influenza A/B-positivity was lower in participants reporting influenza vaccination in the current and previous seasons (odds ratio = 0.55 (95% CI 0.32, 0.95)) versus neither season.

**Conclusions:**

Symptom profiles varied little by aetiology, making distinguishing SARS-CoV-2, influenza and RSV using symptoms challenging. Most symptoms were not explained by these viruses, indicating the importance of other pathogens in syndromic surveillance. Influenza vaccination was associated with lower rates of community influenza test positivity.

**Supplementary Information:**

The online version contains supplementary material available at 10.1186/s12916-024-03351-w.

## Background

Influenza and other respiratory illnesses place large burdens on patients and healthcare [1, 2]. Understanding within-season dynamics is critical to healthcare preparedness and vaccination planning. Routine syndromic and laboratory surveillance is commonly conducted using patients attending community doctors, hospitals, and ambulance services [3], thus being skewed towards symptomatic and more severe cases, and influenced by differential health-care-seeking behaviours [4]. This approach may underestimate the community burden of seasonal influenza, as most cases are mild and/or asymptomatic [5]. Alternative data sources include community surveys, e.g. the UK’s online participatory surveillance system ‘Flusurvey’ [6]. While such cohorts may provide better population-wide estimates, including mild illness, they may still not be representative, tending to underrepresent young children and older adults, both with higher risks of respiratory illness and distinct symptom patterns [7, 8].

Another challenge is the reliance on indicators such as influenza-like illness (ILI) in the absence of virological confirmation [9]. The relationship between ILI and influenza positivity remains complex, influenced by differing case definitions [10, 11], changes in co-circulation of other viruses (notably respiratory syntactical virus (RSV) and SARS-CoV-2) across seasons [12, 13], age-specific dynamics [14], and the non-specific nature of influenza symptoms [15, 16]. Various studies have attempted to assess these relationships, but most have limited their scope to clinical settings, and/or focussed solely on influenza, and/or restricted to patients already reporting ILI or Acute Respiratory Illness (ARI) [7–9, 12–14, 17, 18]. Similarly, influenza vaccine effectiveness evaluation typically uses disease endpoints, rather than protection from infection [19].

Here we use the Office of National Statistics (ONS) COVID-19 Infection Survey (CIS) to investigate the relationship between respiratory infection test positivity and ILI/other self-reported symptoms. This survey differs from sentinel laboratory surveillance in that routine nose and throat swab testing for SARS-CoV-2 (and on a smaller sub-sample, also for influenza A/B and RSV) was conducted on a community cohort, approached at random from address lists, not limited to those contacting healthcare services or with specific case presentations. We estimated SARS-CoV-2, influenza and RSV positivity and incidence across the 2022/2023 winter season, assessed associations between specific symptoms and test positivity, and evaluated the effects of influenza vaccination on positivity.

## Methods

### The ONS COVID-19 Infection Survey (CIS)

CIS was a large longitudinal household survey, broadly representative of the wider UK population (Additional file 1: Supplementary Methods [20]), conducting polymerase chain reaction (PCR) tests for SARS-CoV-2 on self-collected nose and throat swabs and collecting questionnaire data including demographics and symptoms approximately monthly (Additional file 1: Supplementary Methods [21]). The study received ethical approval from the South Central Berkshire B Research Ethics Committee (20/SC/0195). From October 2022, a random subset of ~ 750 swabs received per week were additionally tested by multiplex PCR (ThermoFisher TaqPath™ COVID-19, Flu A/B, RSV ComboKit) in a respiratory pilot study [22]. We analysed swabs taken from 10 October 2022 to 26 February 2023 (≥ 350 respiratory pilot samples/week; ≤ 40 pilot samples/week outside this), when all survey assessments were conducted remotely, either online or by telephone, with swab kits posted to participants and returned by post/courier.

### Self-reported symptoms

Each month, participants were asked whether they had experienced specific symptoms during the last seven days [23]. This analysis included 12 symptoms solicited from the survey start (cough, sore throat, loss of taste, loss of smell, shortness of breath, fever, muscle ache (myalgia), weakness/tiredness (fatigue), headache, nausea/vomiting, abdominal pain, and diarrhoea) and four added September 2021 (wheezing, sneezing, ‘more trouble sleeping than usual’, and ‘loss of appetite or eating less than usual’), but excluded seven unrelated to respiratory illness added January 2022. Influenza-like illness (ILI) was defined using the World Health Organisation (WHO) (concurrent fever and cough) [24] and the European Centre for Disease Prevention and Control (ECDC) (co-presence of ≥ 1 respiratory symptom (cough, sore throat, shortness of breath) and ≥ 1 systemic symptom (fever, fatigue, headache, myalgia)) [25] definitions.

## Statistical methods

### Positivity and incidence

In order to quantify the trends in symptoms and test positivity over time, by age group and overall, we estimated the percentage reporting different symptoms including ILI, and positivity for SARS-CoV-2 (full sample) and influenza A/B and RSV (respiratory pilot only), using negative binomial (log link) Generative Additive Models (GAM) (R *mgcv* package [26]). We used a single explanatory variable for calendar time in days modelled with thin plate splines penalised on the third derivative [27] with *k* = 45 basis functions determining smoothness (approximately total study days(140)/3). Given expected variation, full sample models were run separately for six age groups (2–6SY (school year, ~ 11 years, Additional file 1: Supplementary Methods), 7SY–11SY, 12SY–34, 35–49, 50–64, and 65 +), collapsing to three wider age-groups (2–11SY, 12SY–49, 50 +) for the smaller respiratory pilot. Our focus was on estimating daily trends and how these varied over time: we therefore made a generalisability assumption that the cohort, recruited predominantly from address lists (see Additional file 1: Supplementary Methods) was broadly representative, rather than attempting to use weights (which would have needed to be calculated daily) or post-stratification [28] which could only be done by region, sex, age and ethnicity given lack of available data on the distribution of other factors in the target population. The latter would have required complex interactions between each factor and time which can have convergence problems [21].

In order to estimate incidence from SARS-CoV-2, influenza A/B, and RSV positivity collected in the respiratory pilot study, we used the Richardson-Lucy-type deconvolution. Deconvolution was performed based on daily estimates of test positivity and the distribution of infection (PCR positivity) duration [29, 30], using 10,000 simulations from the posterior GAM distributions (details in Additional file 1: Supplementary Methods). Incidence is presented from 24 October 2022 to 12 February 2023 (weeks 3–18 of the respiratory pilot) as deconvolution tail estimates are highly uncertain. The infection duration was modelled using a Weibull distribution approximating ILI duration for ‘Flusurvey’ respondents [31] (shape and scale parameters to match reported median (9 days) and IQR (reported = 6–15 days, approximated = 5–15 days). Due to insufficient data on appropriate distributions for influenza and RSV in community settings, other infection duration distributions were considered in sensitivity analyses (Additional file 1: Table S1) [32–36].

### Self-reported symptoms, ILI and test positivity

In order to assess the association between self-reported symptoms and test positivity, we estimated a series of GAMs. The probability of testing SARS-CoV-2-positive by age, conditional on reporting specific symptoms, was estimated for the full CIS sample using logistic GAMs. Similar models in the respiratory pilot expanded the outcome to testing positive for influenza A/B, RSV, or SARS-CoV-2, versus no virus identified, using multinomial GAMs (assigning 12 respiratory pilot samples positive for two viruses to the virus with the lowest cycle threshold (Ct) value). Both models included smooths for age and, for SARS-CoV-2 positivity in the larger sample, also calendar time, making predictions at 15 December 2022 to illustrate the contribution of SARS-CoV-2 to reported symptoms when all three pathogens’ positivity was relatively high. We used negative binomial GAMs to estimate the percentage self-reporting ILI and other symptoms by age amongst those testing positive or negative for SARS-CoV-2 in the full sample, and testing positive for influenza A/B and RSV in the respiratory pilot, averaged across the study period. Observations with missing data on self-reported symptoms (< 3%) were excluded from these analyses.

### Influenza vaccination

To assess the effect of self-reported influenza vaccination on influenza A/B, RSV, and SARS-CoV-2 positivity, we used logistic GAMs controlling for demographics (age, sex, household size (1, 2, 3 +), ethnicity (white versus non-white due to small numbers), ever worked in patient-facing healthcare, ever reported long-term health conditions, SARS-CoV-2 vaccination and prior SARS-CoV-2 infection (details in Additional file 1: Supplementary Methods)). All models included smooths for calendar time, age, days since the most recent SARS-CoV-2 vaccination, and days since the start of the most recent SARS-CoV-2 infection (the last two truncated at 365 days (reference category), also with binary variables for unvaccinated or non-infected versus ≥ 365 days). Influenza vaccination was self-reported (“Have you received a flu vaccination since the last assessment” Yes/No/Missing). As the vaccination date was not elicited, participants were considered vaccinated if they had reported an influenza vaccination at a strictly prior assessment, or at the current assessment if the prior assessment was > 45 days ago. Very few participants (< 3%) reported influenza vaccination in 22/23 only (Table 1), so these were categorised in models with “Both 22/23 and 21/22”.

**Table 1:** Study population characteristics

	**Full CIS**	**Respiratory pilot**
**Study visits**		
Observations, *n*	1,352,979	14,939
Positive for SARS-CoV-2, *n* (%)	32,937 (2.4)	354 (2.4)
*Void, n (%)*	25,729 (1.9)	275 (1.8)
Positive for RSV, *n* (%)		181 (1.2)
Positive for Influenza A/B, *n* (%)		130 (0.9)
Symptoms consistent with ILI-WHO, *n* (%)	34,317 (2.5)	367 (2.5)
*Missing, n (%)*	26,936 (2.0)	354 (2.4)
Symptoms consistent with ILI-ECDC, *n* (%)	194,986 (14.4)	2145 (14.4)
*Missing, n (%)*	26,936 (2.0)	354 (2.4)
**Participants**		
Unique participants, *n*	359,186	14,664
Unique households, *n*	185,359	12,554
Observations, median (IQR)	4 (3–4)	1 (1–1)
Country, *n* (%)		
England	301,818 (84.0)	11,748 (80.1)
Scotland	27,625 (7.7)	1390 (9.5)
Wales	19,137 (5.3)	795 (5.4)
Northern Ireland	10,606 (3.0)	731 (5.0)
Sex, *n* (%)		
Female	192,782 (53.7)	7911 (53.9)
Male	166,404 (46.3)	6753 (46.1)
Age group, *n* (%)		
02–6SY	18,484 (5.2)	789 (5.4)
7SY–11SY	21,010 (5.9)	928 (6.3)
12SY–34	41,396 (11.5)	1846 (12.6)
35–49	67,492 (18.8)	2976 (20.3)
50–64	96,229 (26.8)	4080 (27.8)
65 +	114,575 (31.9)	4045 (27.6)
Age, median (IQR)	55 (37–68)	52 (35–66)
Household size		
1	49,334 (13.7)	2151 (14.7)
2	149,214 (41.5)	5621 (38.3)
3 or more	160,638 (44.7)	6892 (47.0)
Ethnicity		
White	332,821 (92.7)	13,402 (91.4)
Non-white	26,365 (7.3)	1262 (8.6)
Ever worked patient-facing health care, *n* (%)	15,447 (4.3)	640 (4.4)
Ever reported long-term health concerns, *n* (%)	86,931 (24.2)	3322 (22.7)
Ever vaccinated against SARS-CoV-2, *n* (%)	334,013 (93.0)	13,586 (92.6)
> 18 years	306,503/310,296 (98.8)	12,308/12,466 (98.7)
Self-reported influenza vaccination		
Both 22/23 and 21/22	215,621 (60.0)	5694 (38.8)
Only 22/23	10,739 (3.0)	269 (1.8)
Only 21/22	56,002 (15.6)	5225 (35.6)
Neither	76,824 (21.4)	3476 (23.7)

## Results

### Trends in test positivity for SARS-CoV-2, influenza, RSV, and self-reported ILI

Between 10 October 2022 and 26 February 2023, the 20-week period when additional influenza/RSV surveillance was conducted and when BQ.1, CH.1.1 and XBB SARS-CoV-2 sub-lineages were co-circulating in the UK, 32,937 (2.4%) of 1,352,979 swab tests conducted at study assessments were SARS-CoV-2-positive (median (IQR) 4 (3–4) tests/participant, 359,186 unique participants) (Table 1). 14,939 (1.1%) randomly selected swabs from 14,664 unique participants were tested in the respiratory pilot, with similar SARS-CoV-2 positivity (*n* = 354, 2.4%). RSV and influenza A/B positivity were lower, 1.2% (*n* = 181) and 0.9% (*n* = 130), respectively. There were 12 (0.08%) coinfections (4 SARS-CoV-2/influenza, 4 SARS-CoV-2/RSV, 4 influenza/RSV; 653 (4.4%) swabs positive for ≥ 1 of the three viruses). Of 130 influenza A/B positives, subtype could be identified from PCR for 87 (remainder too low viral load/high Ct to amplify); 80 (92.0%) were influenza A, 5 (5.7%) influenza B, and 2 (2.3%) both (from whole genome sequencing 8 H1N1, 40 H3N2, and 1 Victoria) [37]. Percentages reporting ILI over the study period were very similar between the respiratory pilot and full CIS sample, with only minor differences in sample demographics (Table 1).

**Fig. 1:**
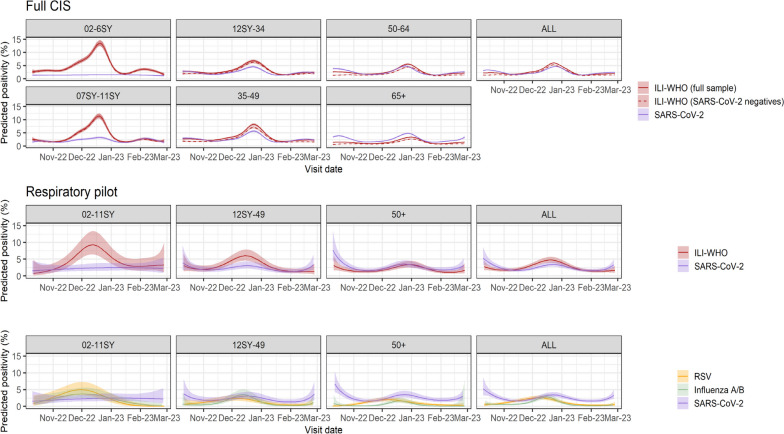
Percentage (95% CI) reporting ILI-WHO (full CIS and respiratory pilot) and test positivity for SARS-CoV-2 (full CIS and respiratory pilot), influenza A/B (respiratory pilot) and RSV (respiratory pilot). Note: SY, school year. See Additional file 1 for raw daily percentages for the full CIS sample (Additional file 1: Fig. S15) and cumulative numbers positive for SARS-CoV-2, influenza A/B and RSV, and reporting ILI-WHO in the respiratory pilot (Additional file 1: Fig. S16)

SARS-CoV-2 positivity and reported ILI-WHO peaked in late December 2022, with similar trends across the pilot and full samples (Fig. 1). Both trends varied by age; SARS-CoV-2 positivity was higher for older versus younger participants, while reported ILI-WHO was higher amongst those in SY11 or younger. In the full sample, SARS-CoV-2 positivity was consistently higher than reported ILI-WHO amongst those ≥ 65 years, and trends in reported ILI-WHO were similar between those testing SARS-CoV-2 negative and positive. RSV and influenza positivity peaked earlier in December 2022, and also varied by age over time, with higher rates in younger children, and earlier peaks in RSV than influenza and SARS-CoV-2, particularly for those ≥ 50 years. Cycle threshold (Ct) values for SARS-CoV-2 followed positivity trends, being lower (i.e. higher viral load) when positivity was higher (Additional file 1: Fig. S1). ILI-ECDC was more common than ILI-WHO, but followed broadly similar trends over time; other symptoms were either approximately constant over time or had similar peaks around December 2022 (Additional file 1: Fig. S2–S4).

### Incidence of SARS-CoV-2, influenza and RSV

**Fig. 2:**
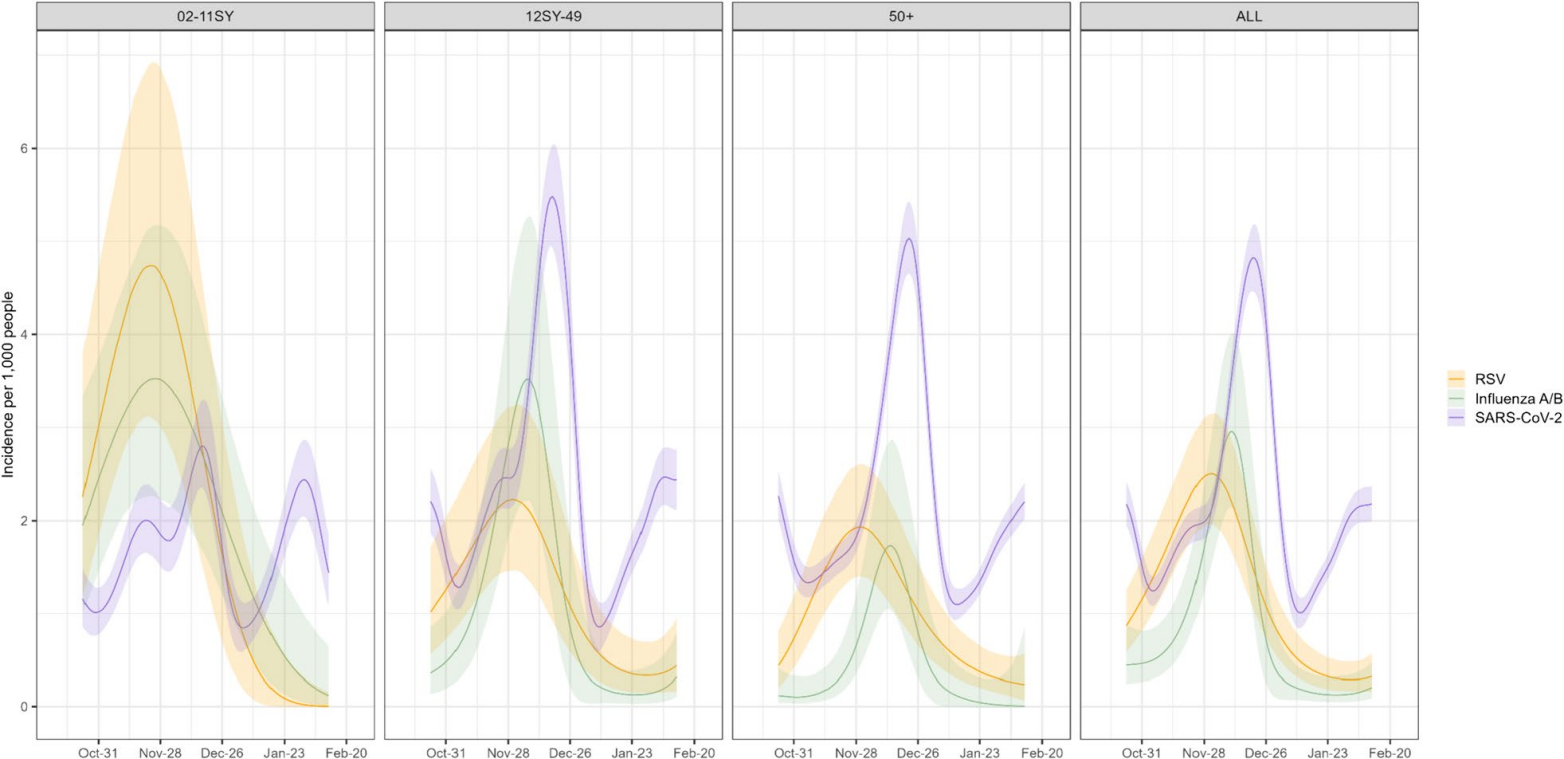
Estimated incidence (95%CI) of SARS-CoV-2 (full CIS), RSV (respiratory pilot), and influenza A/B (respiratory pilot). Note: Time frame covering October 24, 2022–February 13, 2023. SY, school year. Estimates based on a Weibull-ILI survival curve for infection duration. See Additional file 1 for further details on survival distributions (Additional file 1: Table S1, Figure S5)

Estimated incidence of SARS-CoV-2, influenza and RSV therefore also varied by age over time (Fig. 2). In those 2-11SY, peak estimated incidence was higher and occurred earlier for RSV and influenza than SARS-CoV-2 (Table 2), although overlapping credible intervals around estimated incidence over time indicated considerable uncertainty. For older age groups, peak estimated SARS-CoV-2 incidence was higher than RSV and influenza, but with similar shifts in timing (SARS-CoV-2 peaks occurring later than RSV and influenza). However, compared with younger children, peak RSV incidence was lower and slightly later in older age groups (by approximately 1 week), and peak daily influenza incidence also shifted later with increasing age, with 17 days difference between peak influenza incidence between the youngest (2–11SY) and oldest (50 +) age groups (Table 2). The choice of infection duration distribution did not alter the timing of the estimated peaks but influenced absolute incidence estimates (Additional file 1: Fig. S5). Distributions with lower mean duration resulted in higher incidence, by approximately the inverse ratio of means (as expected from first-order approximations), so were ~ 1.4 times higher using a distribution with mean 7.5 versus 10.4 days (Additional file 1: Table S1), although credible intervals overlapped for RSV and influenza**.**

**Table 2:** Estimated peak daily incidence of SARS-CoV-2, Influenza A/B, and RSV, by age group

	**SARS-CoV-2**	**Influenza A/B**	**RSV**
**2–11SY**	2.8 (17 Dec 2022)	3.5 (26 Nov 2022)	4.7 (23 Nov 2022)
**12SY–49**	5.5 (18 Dec 2022)	3.5 (6 Dec 2022)	2.2 (30 Nov 2022)
**50 + **	5.0 (22 Dec 2022)	1.7 (13 Dec 2022)	1.9 (30 Nov 2022)

Incidence estimates are given per 1000 population. Timing of estimated peak incidence is given in parentheses. Full estimates are shown in Fig. 2

### Association between test positivity and self-reported symptoms

**Fig. 3:**
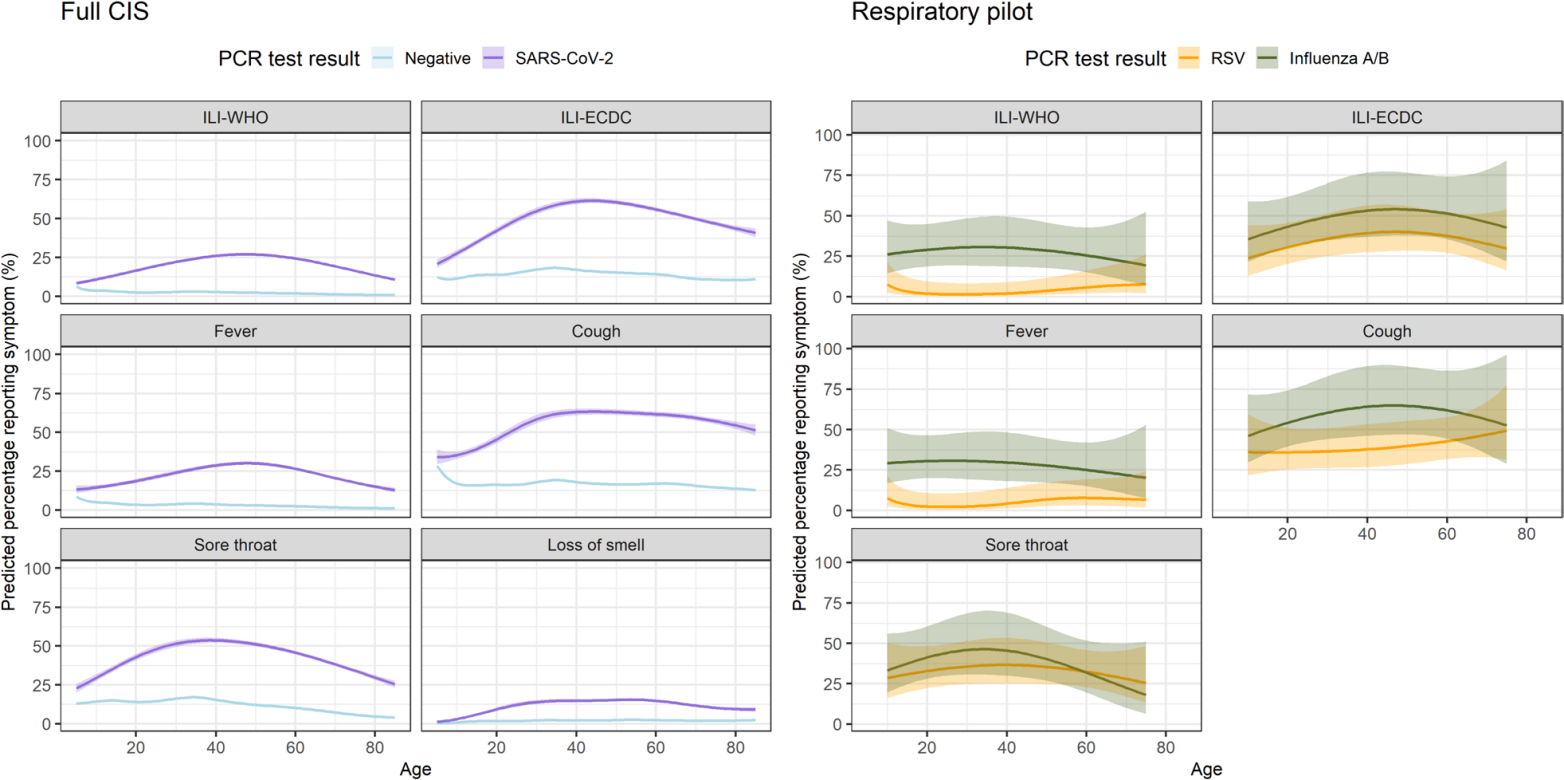
Prevalence of reported symptoms by SARS-CoV-2 test result (full CIS sample), and amongst those testing positive for RSV and influenza A/B (respiratory pilot). Note: See Additional file 1: Fig. S6–S7 for the remaining symptoms. Predictions are averaged across time (no smooth for calendar time included in models). The respiratory pilot analysis excluded loss of smell due to the small absolute number of participants reporting this symptom. Predictions were restricted to ages 10–75 years for the respiratory pilot due to the small absolute number outside this range (approximate 5^th^–95^th^ percentiles), and 5–85 years for the full CIS (approximate 1^st^–99^th^ percentiles)

Considering age as a continuous variable (Fig. 3), over 50% of SARS-CoV-2-positives aged 30–70 years reported symptoms consistent with ILI-ECDC, compared to at most ~ 25% in those 30–65 years for ILI-WHO. ILI-ECDC symptoms were also more commonly reported than ILI-WHO amongst those testing positive for RSV or influenza, with ILI-WHO being particularly uncommon amongst RSV-positives, due to low rates of self-reported fever amongst RSV-positives across all ages. Cough and sore throat were amongst the most common symptoms for SARS-CoV-2-positives, with a prevalence of cough > 50% in those over ~ 20 years. However, in the youngest children, cough was almost as common in SARS-CoV-2-negatives as positives, consistent with multiple other causes. Sneezing, fatigue, and headache were other common symptoms amongst SARS-CoV-2-positives (Additional file 1: Fig. S6), with higher rates amongst middle-aged versus younger and older participants. As for SARS-CoV-2-positives, cough, sore throat, sneezing, fatigue and headache were amongst the most commonly reported symptoms for RSV- and influenza-positives, with broadly similar trends across age (Fig. 3, Additional file 1: Fig. S7), including most symptoms being more commonly reported amongst middle-aged participants. Most symptoms were more commonly reported in influenza- than RSV-positives, wheezing being the main exception, being more commonly reported in older participants testing positive for RSV than influenza or SARS-CoV-2. However, absolute percentages reporting wheezing were lower than for other symptoms, and confidence intervals were wide.

### Association between self-reported symptoms and test positivity

**Fig. 4:**
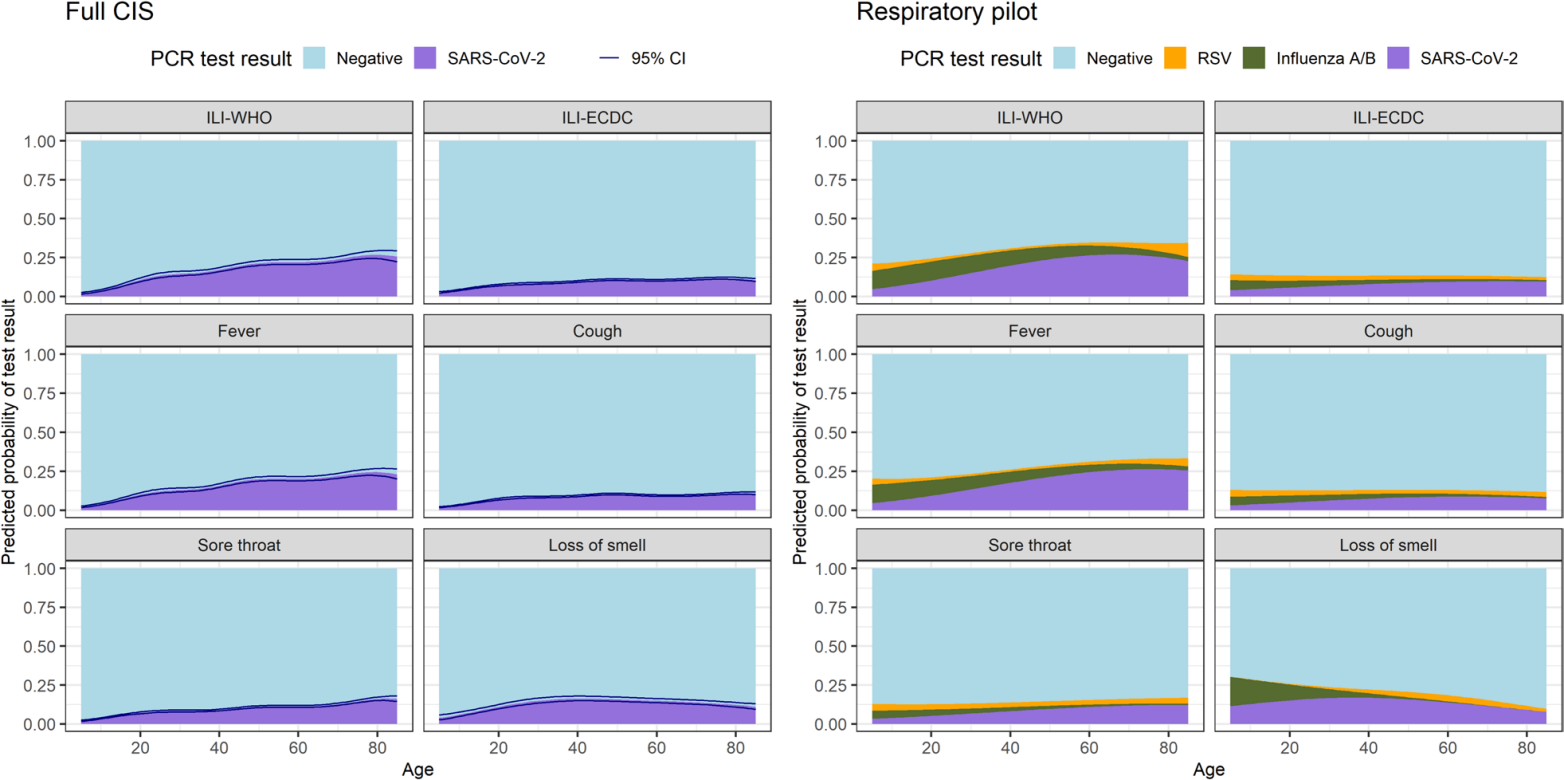
For participants reporting selected symptoms, predicted probabilities of a positive test result for SARS-CoV-2 on 15 December 2022 (full CIS sample), and for SARS-CoV-2, influenza A/B or RSV (respiratory pilot sample), by age. Note: See Additional file 1: Fig. S8–S9, for the remaining symptoms. Predictions for the full CIS sample were made on 15 December 2022 from models which adjusted for time, results for additional dates are shown in Additional file 1: Fig. S17. Predictions for the respiratory pilot are from a model not adjusted for time (given the limited sample size) and therefore represent an overall average over time. Predictions were made for ages 5–85 (approx. 1^st^–99^th^ percentiles)

Nevertheless, whether symptoms were defined by either ILI definition or individually, most (> 65%) symptomatic (community-based) participants were not positive for SARS-CoV-2, influenza A/B, or RSV (Fig. 4, Additional file 1: Fig. S8–S9). The predicted probability of testing SARS-CoV-2-positive given specific symptoms generally increased with age and was higher for ILI-WHO than ILI-ECDC. This appeared to be driven by higher probabilities of SARS-CoV-2 amongst participants reporting fever, the individual symptom with the largest percentage of confirmed viral cases in the full and respiratory pilot samples (Fig. 4). The respiratory pilot estimates suggested that, beyond SARS-CoV-2, RSV and influenza could only explain minor additional fractions of reported symptoms (Fig. 4, Additional file 1: Fig. S9). Further, the probability of confirmed influenza infection tended to decrease with age amongst symptomatic participants, compared to the increasing trend for SARS-CoV-2, although uncertainty was relatively large (Additional file 1: Fig. S10).

### Influenza vaccination

In the respiratory pilot (winter 22/23), influenza A/B positivity was significantly lower for those reporting influenza vaccination both in the current 22/23 and prior 21/22 season versus not reporting influenza vaccination in either season (adjusted OR = 0.55 (95% CI 0.32, 0.95)); there was no evidence of association with influenza vaccination only in the past 21/22 season (aOR = 0.81 (0.52, 1.26), heterogeneity *p* = 0.125) (Table 3, Additional file 1: Fig. S11). Influenza A/B positivity was higher in those working in patient-facing healthcare (aOR = 2.51 (1.31, 4.79)). There was very weak evidence of interaction between vaccination status and age for influenza vaccination in the current and previous seasons (categorising as ≥ versus < 18 years heterogeneity *p* = 0.541 and 0.113, respectively, Additional file 1: Table S2). Including a continuous interaction with age (Additional file 1: Fig. S12), the decreased risk associated with current and previous vaccination was greatest amongst young children and older adults. There was no evidence of association between influenza vaccination and RSV positivity or between prior SARS-CoV-2 infection or vaccination and influenza A/B or RSV positivity (Table 3, Additional file 1: Fig. S11, S13). Interestingly, in the much larger full sample, influenza vaccination in the current and prior season was associated with a slightly elevated risk of SARS-CoV-2-positivity (aOR = 1.10 (1.05, 1.14)), and similarly only in the prior season (aOR = 1.09 (1.05, 1.13)), heterogeneity *p* = 0.688), consistent with competing risks between SARS-CoV-2 and influenza or influenza vaccination targeting those most vulnerable to respiratory infection (Table 3). We found evidence of waning protection against SARS-CoV-2 positivity over time from previous SARS-CoV-2 vaccination, and from previous SARS-CoV-2 infection after the initial period of PCR positivity (Additional file 1: Fig. S14). Those never previously infected with SARS-CoV-2 had a slightly increased risk of SARS-CoV-2 positivity compared to those last infected > 365 days ago (reference category) (aOR = 1.10 (1.06, 1.14)), with risk of reinfection increasing over time from previous test positivity in those last infected < 365 days ago (Additional file 1: Fig. S14). However, those not reporting prior SARS-CoV-2 vaccination had a slightly lower risk compared to those last vaccinated > 365 days ago (reference category) (aOR = 0.81 (0.75, 0.88)).

**Table 3:** Model estimates for influenza vaccination

	**Influenza A/B Respiratory pilot**		**RSV Respiratory pilot**		**SARS-CoV-2 Full CIS**	
	*OR (95% CI)*	*P-value*	*OR (95% CI)*	*P-value*	*OR (95% CI)*	*P-value*
**Flu vaccination 21/22 vs. Neither**	0.81 (0.52, 1.26)	0.359	1.22 (0.83, 1.80)	0.307	1.09 (1.05, 1.13)	< 0.001
**Flu vaccination both 21/22 and 22/23 vs. Neither**	0.55 (0.32, 0.95)	0.032	0.75 (0.46, 1.21)	0.236	1.10 (1.05, 1.14)	< 0.001
**No SARS-Cov-2 vaccination**	1.05 (0.49, 2.23)	0.900	1.45 (0.75, 2.81)	0.268	0.81 (0.75, 0.88)	< 0.001
**No prior SARS-Cov-2 infection**	0.91 (0.53, 1.56)	0.732	0.85 (0.54, 1.35)	0.498	1.10 (1.06, 1.14)	< 0.001
**Upcoming SARS-Cov-2 vaccination in the next 21 days**	-		-		0.44 (0.40, 0.48)	< 0.001
**Female vs. Male**	0.87 (0.61, 1.23)	0.432	0.67 (0.50, 0.90)	0.009	0.94 (0.93, 0.98)	< 0.001
**Ethnicity non-White vs. Ethnicity White**	1.21 (0.72, 2.03)	0.472	0.84 (0.49, 1.44)	0.527	0.88 (0.84, 0.92)	< 0.001
**Household size 2 vs. Household size 1**	1.66 (0.80, 3.44)	0.173	1.11 (0.68, 1.83)	0.671	1.11 (1.07, 1.14)	< 0.001
**Household size 3 + vs. Household size 1**	1.37 (0.64, 2.91)	0.419	0.86 (0.50, 1.47)	0.584	1.13 (1.09, 1.18)	< 0.001
**Ever worked in patient-facing health care**	2.51 (1.31, 4.79)	0.005	1.01 (0.47, 2.17)	0.986	0.94 (0.88, 0.99)	0.029
**Ever reported long-term health concerns**	1.15 (0.72, 1.83)	0.569	1.08 (0.74, 1.57)	0.696	1.02 (1.00, 1.05)	< 0.001

All models include smooths for age, calendar time, days since the most recent SARS-CoV-2 vaccination (truncated at 365 days), and days since the start of the most recent SARS-CoV-2 infection episode (truncated at 365 days). Estimated smooths can be seen in Figs. S15, S16 and S17. The SARS-CoV-2 model also controls for upcoming SARS-CoV-2 in the next 21 days, as individuals testing SARS-CoV-2 positive were asked to defer vaccination (reverse causality), leading to a low probability of vaccination amongst those with a very recent infection. SARS-CoV-2 vaccination coinciding with study visit dates was counted from the next study visit onwards, and flu vaccination coinciding with study visit dates was counted from the next study visit onwards unless the participant had not had any study visits in the past 45 days, in which case vaccination counted from the current visit. Influenza A/B: heterogeneity *p*-value for effects of flu vaccination 21/22 vs. both 21/22 and 22/23 = 0.125. RSV: heterogeneity *p*-value for effects of flu vaccination 21/22 vs. both 21/22 and 22/3 = 0.020. SARS-CoV-2: heterogeneity *p*-value for effects of flu vaccination 21/22 vs. both 21/22 and 22/3 = 0.688

## Discussion

### Positivity and incidence

Estimates from the full ONS CIS and its respiratory pilot suggest that positivity and incidence of SARS-CoV-2, influenza, and RSV varied by age and time across the 22/23 winter season. Peak incidence rates appeared somewhat delayed with increasing age for each virus, but particularly for influenza, with peaks observed approximately 2 weeks later for those 50 years + versus children 11SY or below. RSV peaked before influenza, and then SARS-CoV-2 in each age group, although peaks were relatively close. Increasing influenza cases amongst children could hence provide an early warning for older age groups, consistent with the former being the key driver of influenza transmission [38], and supporting early timing of child vaccination programmes to reduce overall transmission. We also observed higher RSV positivity and incidence for those 2–11SY versus older age groups, and lower influenza positivity/incidence for those ≥ 50 years.

### Symptoms and test positivity

A large fraction of symptoms reported by participants could not be attributed to test positivity for SARS-CoV-2, influenza A/B or RSV. This highlights the role of other infections not included in this study in symptom trends, including rhinovirus, adenovirus, human metapneumovirus, and parainfluenza as identified in syndromic surveillance [39], plus bacterial causes [40]. Given the high prevalence of background symptoms observed in SARS-CoV-2-negatives, the symptoms reported by test positives for SARS-CoV-2, influenza A/B or RSV may not necessarily be caused by these infections specifically. That is, test positives for any of these three infections could even be reporting symptoms that are caused by co-infections with e.g. rhinovirus, rather than by SARS-CoV-2, influenza A/B, or RSV specifically.

RSV-positives generally tended to report fewer symptoms than SARS-CoV-2 or influenza-positives, but symptomatology generally appeared more strongly influenced by age than aetiology. Cough, sore throat, sneezing, fatigue and headache were all amongst the most commonly reported symptoms for each of the three infections, suggesting that discriminating between SARS-CoV-2, influenza and RSV based on symptoms alone may prove challenging, with implications for antiviral treatment and testing. Overall, our findings highlight that in the community, the contributions of these three pathogens to overall symptomatology appear modest. While ILI-ECDC was more commonly reported than ILI-WHO across all ages for the three infections, only ~ 15% of reported ILI-ECDC could be explained by test positivity for SARS-CoV-2, influenza A/B, or RSV. Prior studies have found higher rates of respiratory test positivity amongst those reporting ILI (and conversely, higher rates of ILI amongst influenza-positives), yet these estimates have generally been based on patients presenting to healthcare with symptoms of respiratory infection [8, 9, 12–14, 17, 18, 41]. Such samples will be skewed towards more severe cases, as individuals with milder disease are less likely to seek healthcare. For instance, Casalegno et al. found that 90% of influenza-positives in their study reported cough [8], a considerably higher fraction than our equivalent estimate of ~ 50%, yet this study was restricted to patients presenting to physicians with ARI. On the other hand, a study by Jiang et al. with a comparable design to ours (self-reported symptoms in a community sample regularly tested for influenza A), found that influenza cases accounted for 18% of ILI-ECDC [11], an estimated more in line with our findings.

Careful consideration of background rates and age-specific dynamics are thus necessary when using self-reported symptoms from community cohorts as a surveillance method for respiratory illness, highlighting the potential benefits of more flexible ILI definitions [7, 15]. This finding also underscores the consideration needed when applying ILI definitions in a ‘true community’ context, where the likelihood of milder (and asymptomatic) infection is much higher than in the healthcare settings where surveillance studies are normally performed. Our findings of higher rates of self-reported symptoms in middle-aged participants, broadly consistent across symptoms and the three infections studied, also raise important questions regarding the role of age in infection susceptibility, illness natural history, reporting behaviour, and vulnerability to other symptom-inducing conditions.

Although we confirmed previous findings of high rates of cough in test-negatives [14], we also found evidence of particularly high rates in older RSV-positives. This was the only symptom that approached rates of 50% amongst RSV-positives and confirms prior findings of cough’s relevance to RSV discrimination [12, 42, 43]. In contrast, fever was rarely reported amongst RSV-positives across all ages. Fever has previously been identified as an important predictor of influenza [14, 16, 41], and we also found it was more commonly reported with influenza than SARS-CoV-2 or RSV for those < 20 years. Consequently, fever may have a higher value for predicting influenza in children, yet it was also relatively common amongst SARS-CoV-2-positives. ILI-WHO and ILI-ECDC were similarly reported in SARS-CoV-2-positives and influenza-positives, indicating that the emergence of SARS-CoV-2 may complicate surveillance specifically targeting influenza. As previously suggested, ILI-WHO appears poorly suited to monitor RSV in the community [44–46], due to its inclusion of fever.

### Limitations

Lower specificity and sensitivity of ILI definitions in our community sample compared to those presenting to clinical settings is perhaps unsurprising; however, one limitation is that the approximate monthly testing intervals in the full sample (from which the respiratory pilot was randomly selected) may also have affected the likelihood of symptom reporting, since questionnaires elicited symptoms in the last 7 days. For example, the design will have resulted in cases being identified at differing timepoints in their infection, so that those in a later stage of illness (or experiencing prolonged viral shedding) may appear asymptomatic at assessment although having experienced symptoms earlier in their infection, or may not test positive any longer despite still having symptoms. When positivity rates were low, Ct values supported a larger fraction of cases being identified late in infection (Additional file 1: Fig. S1). Another limitation is that we lacked information on the onset of individual symptoms, as all symptoms experienced within the past week were jointly reported. Further, the likelihood of reporting symptoms consistent with ILI is affected by other demographic factors including gender [47]; we chose to focus on age as the main determinant of symptomatology, determinant of vaccination strategies and hence target of surveillance.

The main limitation is the smaller sample size in the respiratory pilot (which still tested ~ 15,000 swabs), leading to greater uncertainty given the low event rates of RSV and influenza A/B. Although much smaller than the sample tested for SARS-CoV-2, this was still one of the larger community studies to date. Although broadly representative, non-white ethnicities and younger ages remained slightly under-represented, and SARS-CoV-2 vaccination was slightly over-represented (although this has been shown to have short-lived effects on infection). Future studies could try to use modelling techniques such as post-stratification [28] or survey weights to assess the potential impact of disproportional representation of i.e. older ages on positivity and incidence estimates, although these have challenges (see the “Methods” section). Similarly, the limited data on infection duration distributions for RSV and influenza meant incidence estimates were approximate, although the choice of distribution affected absolute levels rather than relative rates or timing of peaks. Furthermore, the 22/23 winter season may not yet equate to steady-state post-pandemic mixing patterns in older adults [48]. Influenza A and B were not differentiated in the multiplex assay, although the vast majority were A on further PCR (only successful in 67%), and we did not consider the impact of SARS-CoV-2 variant on symptomatology. During the study period, BQ.1, CH1.1 and XBB sub-lineages were co-circulating, and the high Ct values (low viral load) of many SARS-CoV-2-positives precluded universal sequencing to identify variants. Nevertheless, prior studies suggest that any symptom differences between influenza A and B are due to age and other risk factors [8, 9].

### Influenza vaccination

We found that influenza vaccination in both the current (22/23) and prior (21/22) seasons was associated with ~ 45% protection against influenza test positivity in this general community sample, with no evidence of the effect of vaccination in the prior season (21/22) only (point estimate ~ 20% reduction). Numbers were too few to robustly assess the impact of vaccination in 22/23 only, although a recent test-negative case–control study suggested this group could have slightly greater benefit [49]. Similarity in influenza strains included in the vaccine across the two seasons means that prior vaccination might have conferred some protection in the 22/23 season [50]. The main influenza strains circulating in the 21/22 and 22/23 seasons were similar, with influenza A (H3N2) being the predominant subtype. In both seasons, the H3N2 strain recommended in the northern hemisphere influenza vaccines (A/Cambodia in 21/22 and A/Darwin in 22/23) also belonged to the same genetic subclade (3C.2a1b) [38, 51]. Alternatively, behavioural patterns or other factors differentiating those choosing vaccination could affect positivity. While live attenuated influenza vaccine (LAIV) could lead to vaccination-induced test positivity in children < 18 years, our estimates of protection were similar restricting to ≥ 18 years, suggesting that effects of vaccination can still be identified in relatively small community cohorts.

## Conclusions

In conclusion, our findings highlight the complex relationship between trends in test positivity for RSV, influenza A/B, and SARS-CoV-2, which peaked successively over the 22/23 winter season but to different degrees in different age groups, and self-reported symptoms. Symptom profiles varied more by age than aetiology, making distinguishing between SARS-CoV-2, influenza and RSV on symptoms alone challenging, and most reported symptoms could not be explained by these viruses. Our findings emphasise the value of community-level data in understanding symptomatology in cases beyond those presenting to healthcare services and have implications for COVID-19 contingency planning, particularly in regards to the percentages not reporting respiratory symptoms.

### Supplementary Information

**Additional file 1: Supplementary Methods. Table S1.** Infection duration distributions. **Table S2.** Differential influenza vaccination effects for Adults/Children. **Figure S1.** Distribution of Ct values. **Figure S2.** Fractions reporting ILI-ECDC and respiratory symptoms. **Figure S3.** Fractions reporting systemic symptoms. **Figure S4.** Fractions reporting loss of taste/smell and GI symptoms. **Figure S5.** Incidence by infection duration distribution. **Figure S6.** Prevalence of symptoms by SARS-CoV-2 test result. **Figure S7.** Prevalence of symptoms for RSV and influenza A/B positives. **Figure S8.** Probabilities of SARS-CoV-2 positivity by symptom. **Figure S9.** Probabilities of SARS-CoV-2, RSV or influenza A/B positivity by symptom. **Figure S10.** Probabilities of SARS-CoV-2, RSV or influenza A/B positivity with 95% Cis. **Figure S11.** Associations between selected variables and influenza A/B positivity. **Figure S12.** Association between age and influenza A/B positivity by vaccination category. **Figure S13.** Associations between selected variables and RSV positivity. **Figure S14.** Associations between selected variables and SARS-CoV-2 positivity. **Figure S15.** Raw percentages reporting ILI-WHO and SARS-CoV-2 test positivity. **Figure S16.** Cumulative numbers of ILI-WHO, SARS-CoV-2, RSV and Influenza A/B. **Figure S17.** Probabilities of SARS-CoV-2 positivity by symptom at different dates [52–57].

## Data Availability

De-identified study data are available for access by accredited researchers in the ONS Secure Research Service (SRS) for accredited research purposes under part 5, chapter 5 of the Digital Economy Act 2017. For further information about accreditation, contact research.support@ons.gov.uk or visit the SRS website.

## Abbreviations

ARI: Acute respiratory illness
CIS: COVID-19 infection survey
Ct: Cycle threshold
ECDC: European Centre for Disease Prevention and Control
GAM: Generative additive models
ILI: Influenza-like illness
ONS: Office of National Statistics
PCR: Polymerase chain reaction
RSV: Respiratory syncytial virus
SY: School year
WHO: World Health Organisation
